# A Multimodal Attract-and-Kill Device for the Asian Citrus Psyllid *Diaphorina citri* (Hemiptera: Liviidae)

**DOI:** 10.3390/insects11120870

**Published:** 2020-12-08

**Authors:** Justin George, Stephen L. Lapointe, Larry T. Markle, Joseph M. Patt, Sandra A. Allan, Mamoudou Setamou, Monique J. Rivera, Jawwad A. Qureshi, Lukasz L. Stelinski

**Affiliations:** 1 Citrus Research and Education Center, Entomology and Nematology Department, University of Florida, 700 Experiment Station Rd., Lake Alfred, FL 33850, USA; justin.george@ars.usda.gov; 2 United States Department of Agriculture, Agricultural Research Service, 2001 South Rock Road, Fort Pierce, FL 34945, USA; larry.markle@ars.usda.gov (L.T.M.); joseph.patt@usda.gov (J.M.P.); 3 18 Valle Vista Dr., Asheville, NC 28804, USA; stephenlapointe@bellsouth.net; 4 Center for Medical, Agricultural, and Veterinary Entomology (CMAVE), Agriculture Research Service (ARS), US Department of Agriculture (USDA), Gainesville, FL 32608, USA; sandy.allan@usda.gov; 5 Kingsville Citrus Center, Texas A&M University, 312 N. International Blvd, Weslaco, TX 78599, USA; Mamoudou.Setamou@tamuk.edu; 6 Department of Entomology, University of California Riverside, Riverside, CA 92521, USA; monique.rivera@ucr.edu; 7 Southwest Florida Research and Education Center, Institute of Food and Agricultural Sciences, University of Florida, Immokalee, FL 34142, USA; jawwadq@ufl.edu

**Keywords:** Asian citrus psyllid, huanglongbing, attract-and-kill device, probing behavior, vision, olfaction, phagostimulant, UV reflectance

## Abstract

**Simple Summary:**

Control of Asian citrus psyllid (*Diaphorina citri*), a vector of *Candidatus* liberibacter asiaticus (*C*Las), contributes to management of citrus greening disease (huanglongbing). We developed two prototypes of a multimodal attract-and-kill (AK) device with specific elements of color, attractant, phagostimulant, ultraviolet (UV) reflectant, and toxicant. Key sensory stimuli comprising the AK ingredients were identified in our current and previous research studies and incorporated into a yellow, slow-release wax matrix (SPLAT). This formulation was applied directly to the surface of yellow cylinders, or to corrugated plastic cards housed within perforated cylinders. Psyllids landing on the devices attempted to feed from the wax matrix, became intoxicated, died, and fell from device surfaces. Our laboratory and field experiments showed that AK devices attracted and killed significantly more adult *D. citri* than ordinary yellow sticky cards and remained fully active over a period of 12 weeks. Effective use of attract-and-kill for management of *D. citri* could reduce need for broad-spectrum insecticide sprays and encourage biological control as part of an integrated approach to huanglongbing (HLB) management in citrus.

**Abstract:**

Phytophagous insects, including Asian citrus psyllids (*Diaphorina citri* Kuwayama), use multiple sensory modalities (vision, olfaction, and gustation,) to locate and accept host plants. We explored incorporation of several sensory cues into a multi-modal attract-and-kill device (AK device) using a three-dimensional shape to increase visibility, as well as elements of color, attractant, phagostimulant, UV reflectant, and toxicant. Attraction of adult *D. citri* to the device was mediated by a combination of a highly reflective yellow cylinder, a UV reflectant compound (magnesium oxide), and an odorant blend as a short-range attractant. The device surface was coated with a slow-release wax matrix (SPLAT™) augmented with a phagostimulant consisting of a 3-component blend (formic acid, acetic acid, and para-cymene) and an insecticide (β-cyfluthrin). Psyllids landing on the device attempted to feed from the wax matrix, became intoxicated, died, and fell from the device. The device remained fully active over a period of 12 weeks partly because dead psyllids or nontargets did not adhere to the surface as occurs on adhesive yellow sticky cards, the industry standard. Laboratory and field assays showed that the device attracted and killed significantly more adult *D. citri* than ordinary yellow sticky cards. This device or a future iteration based on the design elements of this device is expected to contribute to sustainable and environmentally appropriate management of *D. citri* by exploiting the psyllid’s innate behavioral responses to visual, olfactory, and gustatory stimuli.

## 1. Introduction

Citrus greening disease, also called huanglongbing (HLB), is devastating the citrus industry in Florida and worldwide resulting in unprecedented economic loss. Total citrus production in Florida, the state hardest hit by the disease, has declined by approximately 70% since the first detection of HLB in 2004 [1], largely, though not exclusively, due to the rapid spread of HLB to all citrus growing regions of the state. Asian citrus psyllid (*Diaphorina citri* Kuwayama) is the only known insect vector of *Candidatus* Liberibacter asiaticus (*C*Las), the causal pathogen of HLB [2,3]. Suppression of vector populations is considered essential to minimize primary spread to new plantings and secondary spread of the pathogen within established groves [4,5]. Conventional pest and disease management strategies, including intensive and regionally coordinated insecticide applications, have helped with suppression of this vector-pathogen complex; however, disease continues to spread.

Recently, Chow et al. 2019 [6] reported suppression of *D. citri* with an ‘attract-and-kill’ (AK) device that combined visual cues with a pyrethroid toxicant (β-cyfluthrin). Building upon these results, we developed two AK device prototypes that incorporated multiple sensory cues known to affect host finding and probing behaviors of *D. citri*, viz. visual (color, UV reflectant), olfactory (scent lure), and gustatory (phagostimulant) cues. An advantage of AK devices is that they remain clear of insect cadavers, leaf material, or other debris while maintaining an effective kill rate of psyllids over several weeks in the field.

In this study, we used a spreadable emulsified wax dyed with yellow color (‘SPLAT’ ISCA Technologies Inc., Riverside, CA, USA) as the primary carrier and emitter of the sensory stimuli and toxicant material in the AK device prototypes. SPLAT was designed as a slow-release medium for volatile compounds and numerous studies have used SPLAT as a carrier material to measure *D*. *citri* response to various types of test stimuli [7,8,9,10]. A coating of yellow SPLAT was applied to different parts of the AK device prototypes, depending on the prototype’s design (See Section 2).

Because *D. citri* relies on visual cues to locate its host plants [11,12,13,14,15,16], our AK device prototypes utilized visual stimuli as a primary attractant. Attraction of *D*. *citri* to wavelengths perceived as yellow or lime-green in the human visual spectrum has been well documented and is the basis for the use of the “yellow or lime-green” sticky trap for surveillance [11]. In field tests of multiple yellow reflective paints, we found that *D. citri* adults exhibited greater attraction to fluorescent yellow colors than to the bright yellow color used in standard sticky card traps [17]. Therefore, a fluorescent yellow color, shown to be the most attractive to *D*. *citri* in our earlier tests [17], was incorporated into the SPLAT matrix evaluated here.

**Figure 1:**
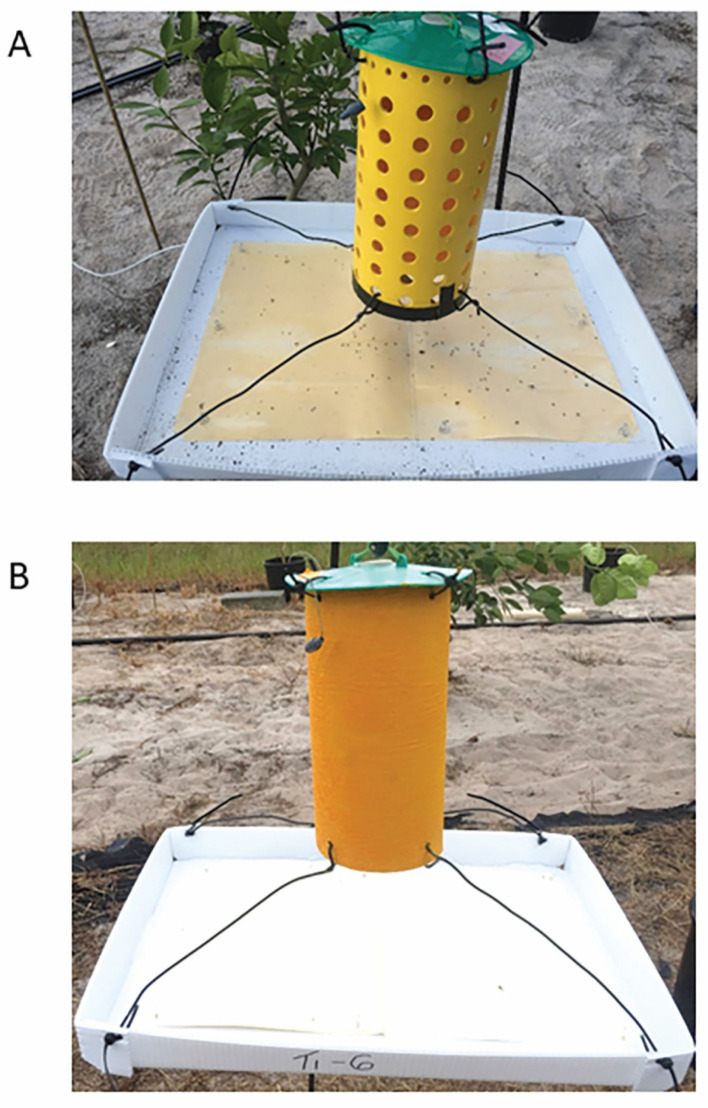
Image of attract-and-kill (AK) device prototypes in the field with horizontal sticky trays to capture dead and moribund *Diaphorina citri.* (**A**) Perforated cylinder AK device with spreadable emulsified wax dyed with yellow color (SPLAT) card inside. SPLAT with all the ingredients was applied to the corrugated plastic card (SPLAT card) inserted inside the cylinder (**B**) Solid cylinder AK device. SPLAT containing all ingredients was applied to the aluminum foil on outer surface of the yellow cylinder. Attractant was released from rubber septa attached to the top of cylinders.

Paris et al. (2017) [16] demonstrated increased attraction of *D. citri* to yellow and green stimuli by incorporating UV reflectants compared with the same colors without UV. When we incorporated a non-toxic UV-reflective compound, magnesium oxide (MgO), into test mixtures of SPLAT, we observed a significant increase in probing by *D. citri* compared with formulations without this ingredient [18]. Based on these results, MgO was included in our SPLAT formulation to maximize both psyllid attraction to and probing on the device surface. In addition, our prototype AK devices had a cylindrical shape to provide a highly visible surface to nearby psyllids and an ample surface area for landing and probing (Figure 1A,B).

Foliar volatiles also play a role in detection, location, and evaluation of potential host plants by *D. citri* [19,20,21,22,23,24,25,26,27]. George et al. [10] found that formic acid and acetic acid, produced as breakdown products of citrus volatiles, elicited strong and consistent antennal responses from *D. citri.* Follow-up studies showed that a mixture of formic and acetic acids, along with a third component, para-cymene, increased psyllid probing into SPLAT [9]. Stylet sheaths deposited by psyllids in the SPLAT containing this three-component blend were significantly more numerous and longer compared to those in other test mixtures, demonstrating that the mixture acted as a phagostimulant [9]. Interestingly, this phagostimulatory effect was especially strong and consistent in *C*Las-infected psyllids [9]. Lastly, a mixture of myrcene, gamma-terpinene, and acetic acid was included as a scent attractant, based on results of investigations that will be published elsewhere (unpublished results). The mixture was dispensed from a rubber septum attached to the AK device prototypes.

## 2. Materials and Methods

### 2.1. Insects Used in Laboratory Assays

Adult *D. citri* were obtained from a colony established in 2000 at the U.S. Horticultural Research Laboratory, Fort Pierce, FL, USA. Psyllids were originally collected from citrus in the field and subsequently reared in greenhouse cages containing orange jasmine, *Murraya exotica* L. (*M. paniculata* auct. non.), and more recently, *C. macrophylla* Wester. The colony was tested quarterly by qPCR according to Li et al. 2006 [28] to confirm absence of *C*Las. All psyllid adults used in the lab cage assays were 7 to 10-days-old.

### 2.2. Attract-and-Kill Device

#### 2.2.1. AK Device Design

The structure of our attract-and-kill device prototypes consisted of a yellow cylinder which provided 360 degrees of visibility to psyllids within their visual range (Figure 1A,B). The cylinders were made from bollard covers (Product no. BC452YN, Crowd Control Store, Dallas, TX, USA), constructed of bright yellow, UV-resistant, high density polyethylene (0.317 cm thick, 12.25 cm OD × 1.625 m), cut into 25 cm long segments.

The cylinders were covered with a convex lid to provide protection from rain and direct sunlight. The lid was the same as used on a standard bucket trap (Great Lakes IPM, Inc., Vestaburg, MI, USA) and was 16 cm in diameter with four 5 mm holes drilled equidistantly around the rim. The lid was attached to the cylinder with twist ties which were affixed through holes in the lid and top of the cylinder (Figure 1A,B).

To collect and quantify psyllids that became moribund or died after exposure to the insecticide, a horizontal tray with sticky panels was suspended below the cylinder (Figure 1A,B). The horizontal tray (61.0 × 45.7 cm) was made from white corrugated plastic sheets (4 mm thick) (Fantastic Displays, Riverside, CA, USA). The tray sides were folded upward creating a 4.5 cm lip to discourage collection of excess foliage and debris and corner holes allowed rain water to drain. The tray was suspended from the cylinder with four coated copper wires (24.6 cm) threaded through holes in the cylinder base.

The sticky panels (47 × 30 cm) were made from manila file folders (Skilcraft, Document Imaging Dimensions, Inc. Binghamton, NY, USA) covered with packing tape (3M Corporate, St. Paul, MN, USA) and then coated on one side with ca. 30 mL Olson Stiky Stuff Coating (Olson Products, Medina, OH, USA) with a foam mini roller. Observations conducted during laboratory and field assays revealed that intoxicated psyllids fell from AK cylinders and could be counted within a 15 cm diameter area beneath the devices, and that most psyllids on the sticky panels were killed by the AK devices rather than the result of incidental captures. Few non-target insects were ensnared by the sticky panels.

#### 2.2.2. Release Formulation

An emulsified wax (SPLAT, ISCA Technologies, Riverside, CA, USA) dyed with yellow (wildfire yellow) color was used as the carrier material for a mixture of insecticide, psyllid attractants, and behavioral modifiers. The attract-and-kill (AK) formulation was applied to different parts of the device depending on the prototype model (see below). The formulation consisted of: (1) a phagostimulant blend to enhance probing activity of the psyllid and thus increase insecticide uptake; (2) magnesium oxide (MgO), a UV reflectant to increase visual attraction, and 3) β-cyfluthrin (12.7% A.I.) (Baythroid XL, Bayer CropScience, Kansas City, MO, USA), a contact insecticide with rapid knock-down activity and which is labelled for use in citrus against *D*. *citri*. The phagostimulant blend [9] was comprised of formic acid (Sigma Aldrich, St. Louis, MO, USA), acetic acid (Sigma Aldrich), and para-cymene (Sigma Aldrich). The amount of active ingredients per 10 g of the AK formulation were 0.1g MgO (1% of mass per 10 g blank SPLAT), 22.8 µL formic acid, 10.7 µL acetic acid, 6.5 µL para-cymene and 0.1 mL β-cyfluthrin (1% of mass per 10 g blank SPLAT). The AK formulation was applied with a paintbrush to create a thin coat of the material. The control AK formulation contained only blank SPLAT.

#### 2.2.3. Scent Attractant

A rubber septum was attached to the side of the cylinder to function as a scent attractant dispenser. The septum was infused with a scent attractant blend (150 µL of a 1:1:1 mixture of myrcene:gamma-terpinene:acetic acid) (unpublished results).

#### 2.2.4. Perforated Cylinder Design

This prototype had a series of holes drilled through the cylinder and a card coated with the AK formulation inserted into the cylinder. The holes permitted psyllids to enter the cylinder and move to the card. We designed this prototype as a means of minimizing non-target species’ exposure to the AK formulation while maintaining a high level of psyllid exposure. Forty-eight holes (12.7 mm diameter) were drilled into each cylinder (Figure 1A). The card inserts (= ‘SPLAT cards’) were cut from sheets of yellow corrugated plastic (Fantastic Displays, Riverside, CA, USA) and had a final dimension of 20 cm Height × 12 cm Length × 4 mm Width. Each SPLAT card was coated with 50 g of AK formulation.

#### 2.2.5. Solid Cylinder Design

The second prototype consisted of a solid cylinder and was designed to maximize exposure of psyllids to the AK formulation. The cylinder was wrapped with a piece of aluminum foil (Reynolds, Louisville, KY, USA) that was covered with 80 g of AK formulation (Figure 1B).

### 2.3. Screening of Insecticides by Evaluating Psyllid Mortality

We predicted that fast acting insecticides with quick knockdown properties would be needed to effectively kill alighting and probing psyllids on our attract-and-kill device under field conditions. However, a variety of modes of action were tested to facilitate possible future registration in citrus and to eliminate candidates that could be incompatible with our chosen SPLAT carrier and other ingredients. Insecticides tested included pyrethroids (Baythroid XL, Tombstone; Bayer CropScience, Kansas City, MO, USA), a ryanoid (Exirel; Dupont, WA, USA), repellent/sufficant (Azatin; OHP Inc., Bluffto, SC, USA), and spinosoids (Spinosad; Spinosyn A & D; Dow Agroscience, Indianapolis, IN, USA). Each insecticide was added at the rate of 1mL/100 gm of SPLAT (1% by weight). Baythroid XL (β-cyfluthrin) and Tombstone (cyfluthrin) were also tested at a higher 2% rate. Insecticides were chosen based on known efficacy against *D*. *citri* and registration for use in citrus against *D. citri*. Fully prepared AK devices with various SPLAT formulations containing each insecticide (*n* = 3) were tested in our cage assays after periods of aging under laboratory conditions to assess residual activity for up to 14 weeks. Insecticides that caused ≥50% mortality of exposed psyllids during the initial 4 h of laboratory cage assays were investigated further to determine residual activity.

#### Laboratory Cage Assays

Insecticides were tested under conditions similar to rearing described above to quantify psyllid mortality. Insecticides were added to the complete AK formulation at 1% w/w of formulated product. Control devices received blank yellow SPLAT. AK formulations were applied to plastic cards or solid cylinders (Figure 1B) suspended inside a mesh cage (75 × 75 × 75 cm). One hundred *D. citri* adults starved for 2 h prior to assays were released on the floor of each cage. Mortality of *D. citri* was quantified at 1, 2, 4, and 20 h after release. After the 20 h exposure (day 1), AK devices were removed and placed in a laboratory exhaust hood for aging. Devices were tested weekly to measure residual activity. There were three replicates per treatment (3 separate devices per prototype per insecticide).

### 2.4. Field Evaluation of Attract-and-Kill Device Prototypes in Potted Citrus

The two prototype AK devices were evaluated in field trials using potted citrus plants. Attraction of *D. citri* to the two AK devices and psyllid mortality were evaluated for 14 days following device deployment. Experiments were performed as paired tests in which each AK treatment was deployed with a paired 20 × 12.5 cm yellow sticky card (Great Lakes IPM Inc., Vestaburg, MI, USA) placed 0.5 m away from the AK devices that served as a control for assessing psyllid attraction to each device. Psyllids killed by AK devices were quantified using horizontal sticky trays hung beneath devices as described above.

Experiments were conducted at the USDA experimental farm in Fort Pierce, FL using potted two-year old, non-bearing citrus (Valencia on Swingle rootstock) ca. 1.5 m in height. A total of 120 trees were obtained from Southern Citrus Nurseries, Dundee, Florida in August of 2018 and confirmed free of *C*Las as described above. Plants were arranged in 4 rows at 2.4 m spacing between trees and supplied with microjet irrigation. Prior to initiation of the experiment, plants were trimmed to induce growth of new leaf flushes. Two thousand laboratory reared adult *D. citri* were then released at the site 10 d prior to the experiment. The experiment was set up as paired treatments. Each AK prototype paired with a yellow sticky card control placed 0.5 m away was replicated 6 times. AK treatments were arranged 18 m apart in each row of trees. Psyllids found underneath AK devices or adhering to yellow sticky cards were counted 3, 7, and 14 d after deployment of the devices. The cumulative number of *D. citri* killed per device or found on paired control sticky cards was calculated on day 14. Data were analyzed by paired *t*-test using JMP (SAS, Cary, NC, USA).

### 2.5. Evaluation of Attract-and-Kill Prototypes in a Commercial Citrus Grove

The two prototype AK devices were evaluated in a commercial citrus grove consisting of a mixed planting of 4-year-old bearing (ca. 2.5 m^3^ canopy volume) and 1.5-year-old non-bearing (~1 m^3^ canopy volume) citrus (*Citrus sinensis* L. Osbeck v. Valencia on Swingle rootstock) with 2.4 m in-row and between-row spacing at Lake Wales, FL. Field experiments were performed during the summer and fall of 2019 and coincided with peak psyllid activity. This experiment location received minimal insecticide sprays for psyllid control throughout the year and had well-established *D. citri* populations throughout the course of the experiment as determined by tap sampling.

In the first experiment, the perforated cylinder AK devices were deployed in non-bearing trees. Solid cylinder AK devices were deployed in mature trees. A given AK device prototype and a corresponding yellow sticky card control were arranged on trees next to each other (2.4 m apart) as paired treatments to reduce statistical noise due to variability in within-grove psyllid populations. Pairs of AK device and yellow sticky card were arranged 20 trees apart (48 m) in the same row and were replicated six times in two tree rows. The number of dead *D. citri* adults on horizontal sticky trays beneath AK devices and paired yellow sticky cards were monitored weekly. Yellow sticky cards were replaced every two weeks as they became fouled with debris. Psyllid kill was monitored for 11 or 12 weeks.

A second experiment was performed in which the solid cylinder AK devices were evaluated in non-bearing trees at the Lake Wales location, while perforated cylinder devices were evaluated in mature trees. Device preparation, experimental design, and data collection were identical to that described for the first experiment. Data were analyzed by paired *t*-test using JMP statistical program (SAS, Cary, NC, USA).

### 2.6. Evaluation of Incidental D. citri Catch on Horizontal Sticky Trays Below AK Devices

An experiment was conducted to estimate the proportion of *D. citri* catches on horizontal sticky trays resulting from attempted feeding or contact with the AK devices versus the proportion of psyllids possibly attracted to and caught on the horizontal trays alone. The experiment was performed in a commercial citrus grove consisting of 4-year-old bearing (~2.5 m^3^ canopy volume) citrus (*C. sinensis* L. Osbeck v. Valencia) planted using 2.4 m in-row and between-row spacing at Lake Alfred, FL. Field experiments were performed during the summer of 2020 and coincided with peak psyllid activity. This experiment location received minimal insecticide sprays for psyllid control annually and had a high population of *D. citri* as determined by tap sampling.

In this experiment, only the solid cylinder AK devices were deployed. The three paired treatments were as follows: AK device + horizontal tray with sticky liner, horizontal tray with sticky liner only, and yellow sticky card. Device preparation and experimental design were identical to that described for the first experiment. Treatment sets were replicated six times. Psyllid catch was recorded weekly for eight weeks. Overall mean differences in *D. citri* catch between treatments were analyzed by ANOVA followed by Tukey’s HSD, using JMP statistical program (SAS, Cary, NC, USA).

## 3. Results

### 3.1. Screening of Insecticide Classes

**Figure 2:**
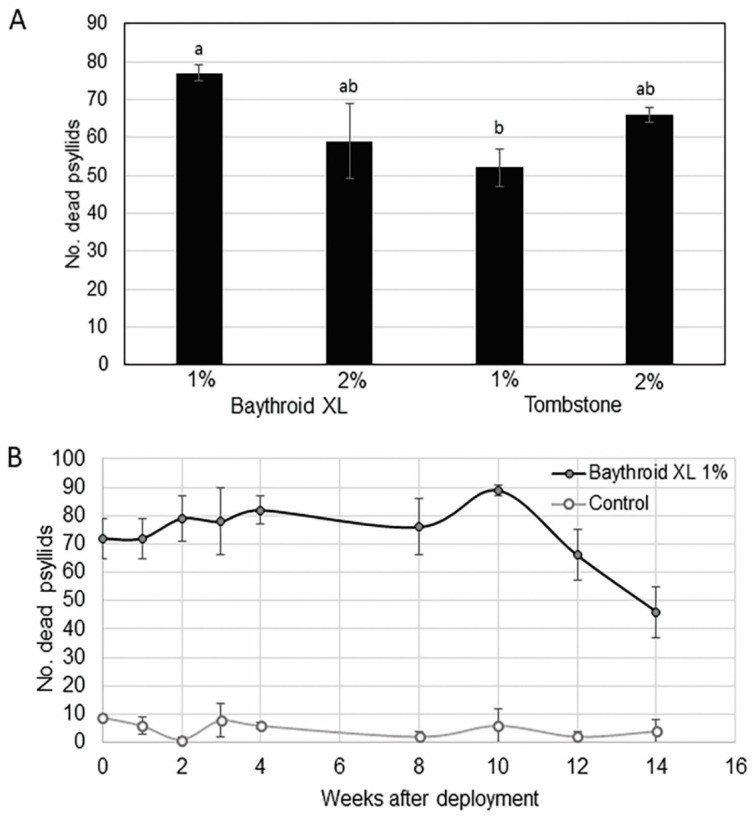
(**A**) Mean (±SEM, *n* = 3) capture of dead or moribund *Diaphorina citri* adults from attract-and-kill devices baited with SPLAT containing two pyrethroid insecticides at two concentrations by volume 4 h after exposure. Means labelled with different letters are significantly different by Tukey’s HSD after a significant ANOVA (α = 0.05). (**B**) Mean (±SEM, *n* = 3) capture of dead or moribund *Diaphorina citri* adults from attract-and-kill devices baited with SPLAT containing Baythroid XL insecticide (1% β-cyfluthrin) compared with similar devices baited with SPLAT without insecticide.

Mortality of *D. citri* released in cages with AK devices was greatest when pyrethroids were incorporated into the formulation as the toxicant. Only pyrethroid insecticides caused >50% psyllid mortality within 4 h (Table 1). Of the pyrethroid treatments tested, β-cyfluthrin (Baythroid XL) at a 1% rate by weight caused significantly higher mortality (*F*_3,16_ = 3.75; *p =* 0.03, *n* = 12) than the other pyrethroid treatments, irrespective of loading rate (Figure 2A). Significantly more *D. citri* were killed by 1% β-cyfluthrin (Baythroid XL) than the blank control after 4 h (Table 1). Increasing the amount of β-cyfluthrin two-fold (2%) did not significantly increase psyllid mortality. Cyfluthrin (Tombstone), at 1 and 2% rates by weight, also caused significantly higher mortality of *D. citri* (*p* < 0.0001) than controls.

Baythroid XL was tested further to determine residual activity of this toxicant for up to 14 weeks after aging under laboratory conditions. AK devices treated with 1% Baythroid XL killed >50% of *D. citri* up to 12 weeks after application (Figure 2B) in cage assays. Significantly more *D. citri* (*p* < 0.05 *n* = 3) were killed by AK devices with Baythroid XL (1%) than the control for up to 14 weeks of laboratory hood aging (Figure 2B). The mortality of released *D. citri* in cages with AK devices treated with Baythroid XL (1%) dropped to 46% by 14 weeks of aging. All other insecticides tested caused very low psyllid mortality during the 4 h bioassay (Table 1).

### 3.2. Evaluation of Attract-and-Kill Device in Potted Citrus under Field Conditions

**Figure 3:**
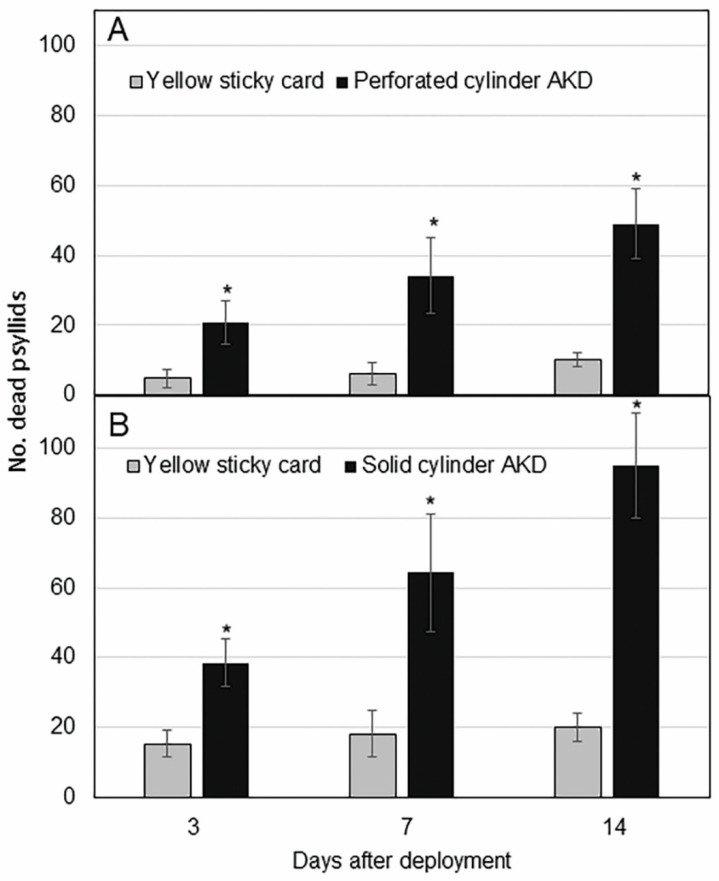
(**A**) Mean (±SEM, *n* = 6) number of *Diaphorina citri* adults attracted to and killed by perforated cylinder AK device compared to that captured on yellow sticky cards 3, 7, and 14 days after deployment. (**B**) Mean (±SEM, *n* = 6) number of *Diaphorina citri* adults attracted to and killed by solid cylinder AK device compared to that on yellow sticky cards 3, 7 and 14 days after deployment. Means labelled with asterisks are significantly greater than their paired control traps by paired *t* test (α = 0.05, *n* = 6).

Significantly more *D. citri* were killed by perforated cylinders with internal SPLAT cards than were counted on paired sticky cards on day 3 (*p* = 0.04), 7 (*p* = 0.005) and 14 (*p* = 0.004) (Figure 3A). Psyllids were found directly below the perforated cylinders, indicating that psyllids alighted on the internal cards and became intoxicated following contact with the AK formulation. This prototype also killed approximately five times more *D. citri* than were captured on yellow traps after two weeks.

Significantly more dead *D. citri* were found beneath solid cylinder devices than on companion sticky cards on day 3 (*p* = 0.014), 7 (*p* = 0.005), and 14 (*p* < 0.0001) (Figure 3B). Approximately five times more psyllids were found killed beneath solid cylinder AK devices than were captured on paired yellow sticky card controls by day 14. We observed that after *D. citri* landed on AK devices, they became intoxicated and quickly fell to the sticky pane beneath the device, indicating fast knockdown due to the pyrethroid.

### 3.3. Evaluation of Attract-and-Kill Device in a Commercial Citrus Grove

**Figure 4:**
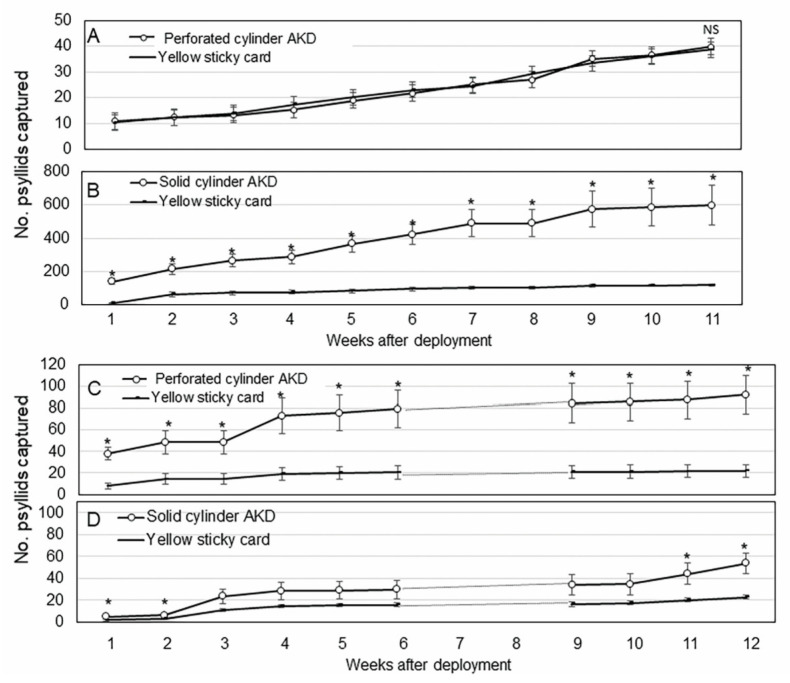
(**A**) Mean (±SEM, *n* = 6) cumulative number of *D. citri* adults captured by perforated cylinder AK device over 11 weeks in young citrus trees. (**B**) Mean (±SEM, *n* = 6) cumulative number of *Diaphorina citri* adults captured by solid cylinder AK devices over 11 weeks in mature citrus trees. (**C**) Mean (±SEM, *n* = 6) cumulative number of *D. citri* adults captured by perforated cylinder AK device during 12 weeks in mature citrus trees. (**D**) Mean (±SEM, *n* = 6) cumulative number of *D. citri* adults captured by solid cylinder AK device over weeks 12 weeks in young citrus trees. Data were not collected on weeks 7 and 8 due to hurricane Dorian. Means labelled with asterisks are significantly greater than their paired sticky card traps by paired *t-*test (α = 0.05).

There was no statistical difference between the cumulative number of dead *D. citri* found beneath perforated cylinder AK devices and those counted on yellow cards in the young, non-bearing trees (*p =* 0.80, *n* = 6) (Figure 4A). Significantly more *D. citri* were captured beneath solid cylinder devices deployed in mature trees than were captured on standard yellow sticky cards. More *D. citri* were killed by solid cylinder AK devices (140 ± 15) than found on control yellow sticky cards (10 ± 3) after 1 wk of deployment (*p <* 0.0001, *n* = 6). The mean cumulative number of *D. citri* killed by solid cylinder AK devices was significantly higher (*p =* 0.002, *n* = 6) (597 ± 95) and almost six times greater than that counted on paired yellow sticky cards (118 + 12) during the 11 weeks test period in mature trees (Figure 4B). The numbers of *D. citri* captured beneath AK devices were significantly higher during wk 1 (*p* < 0.001), 3 (*p* < 0.001), 4 (*p* < 0.05), 5 (*p* < 0.001), and 7 (*p* < 0.05) than that counted on the paired control yellow sticky cards. The cumulative number of *D. citri* killed by AK devices was significantly higher than that counted on yellow sticky cards during all eleven weeks of the experiment (*p* < 0.001, *n* = 6) (Figure 4B). Cumulatively, approximately 50% of *D. citri* killed by AK devices were collected during the initial 4 weeks after deployment and 80% of the cumulative total was collected by 7 weeks.

Significantly more *D. citri* were killed by perforated cylinder AK devices (92 ± 18) than were counted on control sticky cards (22 ± 6) in mature trees during the 12 week test period (*p =* 0.003, *n* = 6) (Figure 4C). Overall, fewer *D. citri* were trapped in the young trees (40 ± 4) than mature trees (92 ± 18) which may have affected outcomes (Figure 4A,C). In the younger, non-bearing trees, significantly more *D. citri* were killed by solid cylinder AK devices (54 ± 10) than were found adhering to paired control sticky cards (23 ± 3) (*p =* 0.001, *n* = 6) (Figure 4D). The total number of *D. citri* killed by solid cylinder AK devices in mature trees (Figure 4B) was eleven-fold greater than that killed by these same devices in young trees (Figure 4D). Significant rain associated with Hurricane Dorian occurring in weeks 7 and 8 (3–10 September 2019) may have affected psyllid numbers during this experiment.

### 3.4. Evaluation of Incidental D. citri Catch on the Horizontal Sticky Tray below AK Device

**Figure 5:**
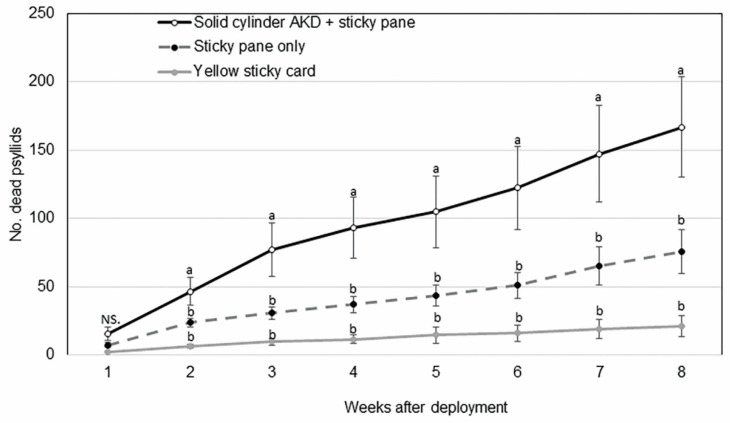
Mean (±SEM, *n* = 6) cumulative number of *Diaphorina citri* adults captured on horizontal trays below solid cylinder AK devices, horizontal trays alone, and yellow sticky cards over eight weeks in 4-year-old citrus trees. Weekly comparison of means labelled with different letters are significantly different by Tukey’s HSD after a significant ANOVA (α = 0.05).

The mean (±SEM, n = 6) cumulative number of *D. citri* killed on trays suspended beneath solid cylinder AK devices (167 ± 36) was significantly higher than that found on horizontal sticky trays alone (76 ± 16) or control yellow sticky cards (21 ± 7) after 8 weeks of deployment (*p* < 0.005) (Figure 5). The mean number of *D. citri* killed by AK devices was significantly higher than that counted on yellow sticky cards during all the eight weeks of the experiment (*p* < 0.05, n = 6). Significantly more adults were counted on sticky trays beneath AK devices than on horizontal sticky trays alone during the eight weeks of the experiment (*p* < 0.05, n = 6). Weekly tap sampling showed no differences in *D. citri* populations on citrus trees between treatments throughout each week of the experiment.

## 4. Discussion

Two types of prototype AK devices were developed and tested. Prototype 1 consisted of a perforated cylinder with a card coated with the AK formulation placed inside (Figure 1A). This prototype was designed to prevent exposure of non-target insects to the toxicant and took advantage of the behavioral tendency of *D. citri* to crawl and hide within tree branch grooves. Prototype 2 consisted of a solid yellow plastic cylinder coated with the AK formulation. This increased the lethal surface area of the devices and attracted and killed approximately five times more *D. citri* adults than the first prototype in field experiments. The second prototype was effective for up to 12 weeks in field tests although its surface received greater exposure to UV radiation and rainfall than that of the perforated cylinder with internal card.

After alighting on devices, psyllids probe their surface with their piercing/sucking mouthparts and acquire toxicant from the formulation. A 3-component phagostimulant blend (3.5:1.6:1 blend of formic acid: acetic acid: para-cymene) identified and optimized in our previous research [9] was incorporated into the SPLAT matrix to induce more and longer probes. Addition of the phagostimulant blend with MgO increased psyllid probing activity and mortality on insecticide-treated SPLAT by 15–20% compared with either cue deployed individually [18].

Attract-and-kill formulations typically require fast-acting contact insecticides to eliminate insects attracted to the target [6,29]. A previously developed AK device for *D. citri*, which consisted of polyvinyl chloride (PVC) impregnated with the pyrethroid β-cyfluthrin caused rapid knockdown mortality of *D. citri* with prolonged residual toxicity [6]. We evaluated several insecticide chemistries and only pyrethroids (β-cyfluthrin and cyfluthrin) caused both significant mortality of *D. citri* and exhibited long-lasting residual activity with the devices tested here (Table 1). We judged Baythroid XL (β-cyfluthrin) to be the most effective. We observed that *D. citri* adults alighting on devices probed the SPLAT matrix containing Baythroid XL, becoming intoxicated and rapidly knocked down from devices (Figure 2A,B).

In field experiments using potted Valencia plants, both AK device prototypes were more effective in attracting and killing psyllids than the commercial standard yellow sticky card (Figure 3A,B). The solid cylinder, coated with SPLAT, killed five times more *D. citri* than were captured by yellow sticky cards. This solid cylinder also killed twice as many psyllids as the perforated cylinder. These results suggest that *D. citri* made more contact with the AK formulation on the surface of the solid cylinder than on the internal SPLAT card within the perforated cylinder.

The non-adhesive design of our devices presents a problem in evaluating their efficacy in the field as dead or moribund psyllids fall from the device and can be difficult to find and count. This attribute also contributes to the longevity of the device since the surface does not become fouled with dead psyllids or nontarget organisms. To quantify psyllids killed after encountering AK devices, we fashioned horizontal adhesive trays and fastened them below devices to catch psyllids as they fell off. That raised a concern that the trays themselves, not envisioned as part of an eventual product, were contributing to psyllid catch. While the trays with sticky liners did catch *D. citri* adults, the numbers were significantly lower than the numbers caught by the AK devices fitted with trays (Figure 5). We interpret this as further proof that the AK design was effective and, by itself, outperformed the traditional yellow sticky card over several weeks. It should also be noted that the yellow sticky cards were replaced periodically through the trial as their surfaces became occluded by insects and debris. The AK devices were not replaced for the duration of the field trials. Of the two AK prototypes tested, the solid cylinder was most effective in both non-bearing and mature citrus (Figure 4B,D).

Adult *D. citri* rely on several sensory modalities for host finding that can be targeted to develop management tools. AK has been developed for other citrus [30] and fruit [31,32,33] pests. The concept for an AK device for *D. citri* reported here was constructed of inexpensive components that could be mass-produced with existing technology. Evolution of this design is ongoing as we attempt to include all elements of the design into simpler, cheaper, and easier to deploy devices for commercial citrus groves, dooryard citrus, and enclosed production facilities. An effective AK device for management of *D. citri* could reduce use of broad-spectrum insecticides and encourage biological control as part of an integrated approach to HLB management in citrus.

## 5. Conclusions

Visual, olfactory, and gustatory cues were combined to develop an attract-and-kill device that was attractive and lethal to *D. citri*. The device made use of a previously identified phagostimulant, which was combined with a fast-acting insecticide into a delivery vehicle, SPLAT. This device employed a non-sticky surface containing toxicant and was therefore non-fouling and capable of deployment within tree canopies without affecting non-targets. This device attracted and killed more *D. citri* than were captured by standard yellow sticky traps and could also be adapted for psyllid monitoring to enable need-based management decisions. Our current efforts are focused on optimizing deployment rates of devices per area of crop, as well as, efficacy comparisons with standard psyllid suppression programs employing calendar-based insecticide sprays. Our goal is to incorporate AK devices into HLB management programs to decrease reliance on calendar-based sprays and their associated non-target consequences.

## Figures and Tables

**Table 1:** Mean (±SEM, *n* = 3) mortality of *Diaphorina citri* after 4 h exposure to insecticides in SPLAT. Data analyzed by paired *t*-test, *df* = 6 (α = 0.05).

Insecticide Tested	Mortality (%)		*t-ratio*	*p* Value
	Insecticide	Control		
Baythroid XL 1%	77 ± 1.9	6 ± 1.7	27.76	<0.0001
Tombstone 2%	66 ± 1.9	2 ± 0.6	10.72	<0.0001
Baythroid XL 2%	59 ± 9.6	6 ± 1.8	4.89	0.002
Tombstone 1%	52 ± 4.6	2 ± 1.1	10.47	<0.0001
Spinosad 2%	7 ± 2.6	2 ± 0.7	1.87	0.11
Exirel 2%	4 ± 0.3	3 ± 0.8	1.10	0.315
Azatin 2%	3 ± 1.8	4 ± 1.9	0.08	0.94
